# The GPCR properties of polycystin-1- A new paradigm

**DOI:** 10.3389/fmolb.2022.1035507

**Published:** 2022-11-04

**Authors:** Robin L. Maser, James P. Calvet, Stephen C. Parnell

**Affiliations:** ^1^ Department of Biochemistry and Molecular Biology, University of Kansas Medical Center, Kansas City, KS, United States; ^2^ Department of Clinical Laboratory Sciences, University of Kansas Medical Center, Kansas City, KS, United States; ^3^ Jared Grantham Kidney Institute, University of Kansas Medical Center, Kansas City, KS, United States

**Keywords:** ADPKD, polycystin-1, polycystin-2, receptor-ion channel complex, GPS cleavage, tethered peptide agonist, heterotrimeric G proteins

## Abstract

Polycystin-1 (PC1) is an 11-transmembrane (TM) domain-containing protein encoded by the *PKD1* gene, the most frequently mutated gene leading to autosomal dominant polycystic kidney disease (ADPKD). This large (> 462 kDal) protein has a complex posttranslational maturation process, with over five proteolytic cleavages having been described, and is found at multiple cellular locations. The initial description of the binding and activation of heterotrimeric Gαi/o by the juxtamembrane region of the PC1 cytosolic C-terminal tail (C-tail) more than 20 years ago opened the door to investigations, and controversies, into PC1’s potential function as a novel G protein-coupled receptor (GPCR). Subsequent biochemical and cellular-based assays supported an ability of the PC1 C-tail to bind numerous members of the Gα protein family and to either inhibit or activate G protein-dependent pathways involved in the regulation of ion channel activity, transcription factor activation, and apoptosis. More recent work has demonstrated an essential role for PC1-mediated G protein regulation in preventing kidney cyst development; however, the mechanisms by which PC1 regulates G protein activity continue to be discovered. Similarities between PC1 and the adhesion class of 7-TM GPCRs, most notably a conserved GPCR proteolysis site (GPS) before the first TM domain, which undergoes autocatalyzed proteolytic cleavage, suggest potential mechanisms for PC1-mediated regulation of G protein signaling. This article reviews the evidence supporting GPCR-like functions of PC1 and their relevance to cystic disease, discusses the involvement of GPS cleavage and potential ligands in regulating PC1 GPCR function, and explores potential connections between PC1 GPCR-like activity and regulation of the channel properties of the polycystin receptor-channel complex.

## 1 Background

### 1.1 PKD genes and centrality of polycystin-1

Autosomal dominant polycystic kidney disease (ADPKD) is caused by mutations of the *PKD1* or *PKD2* genes, which encode the proteins polycystin-1 (PC1) and polycystin-2 (PC2), respectively. PC1 and PC2 are integral membrane proteins proposed to co-exist as a heterotetrameric receptor-like/ion channel complex. Both proteins are found in multiple cellular locations, including the ER, plasma, and primary ciliary membranes. Together, PC1 and PC2 are thought to play an important role in cellular ion homeostasis and signal transduction, possibly in response to ligand binding and mechanical stimuli (Nigro and Boletta, 2021).

Mutations in additional genes have also been reported that account for a small fraction of ADPKD cases. These genes and their protein products include *GANAB/*glucosidase II alpha subunit, *DNAJB11/*DnaJ homolog (hsp40) subfamily B member 11, *ALG9/*alpha-1,2-mannosyltransferase, and *IFT140/*intraflagellar transport 140 (Cornec-Le Gall et al., 2018a; Lemoine et al., 2022). The *GANAB* and *DNAJB11* products are ER-resident proteins involved in protein transport, folding and quality control. The *IFT140* protein is in a complex responsible for retrograde transport in the primary cilium and is involved in ciliary entry of GPCRs. Of the ADPKD genes, mutation of *PKD1* is by far the most predominant cause of the disease (∼78%), followed by mutation of *PKD2* (15%) and *IFT140* (∼2%) (Senum et al., 2022). Interestingly, the protein products of *PKD2*, *GANAB*, *DNAJB11*, and the genes *SEC63* and *PRKCSH*, which are mutated in autosomal dominant polycystic liver disease, are necessary for the proper biogenesis or trafficking of PC1 (Fedeles et al., 2011; Besse et al., 2017; Cornec-Le Gall et al., 2018b; Besse et al., 2019; Hu and Harris, 2020). Such findings reveal the key importance of PC1 in the pathogenesis of the cystic diseases caused by each of these genes and underscore the need to better understand the structure-function relationships and the central role of this complicated protein.

### 1.2 The structural and functional complexity of Polycystin-1

**FIGURE 1:**
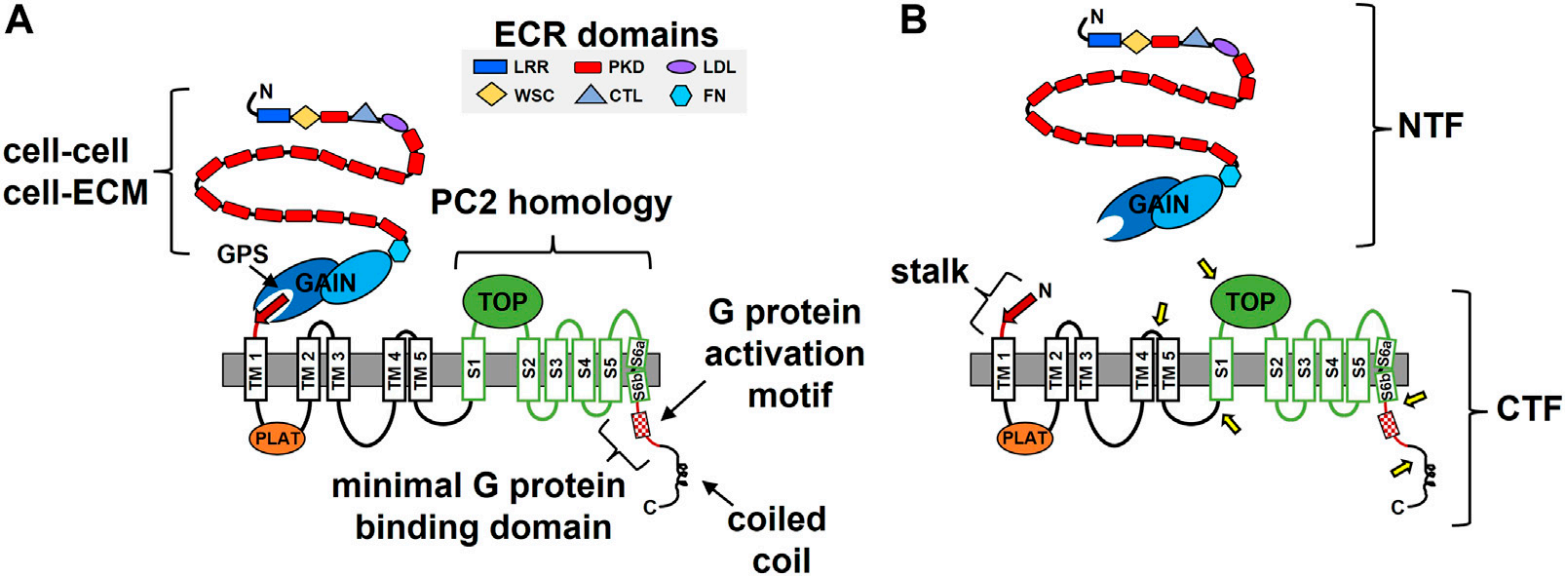
The structure-function features of polycystin-1 (PC1). **(A)** Domains identified in the N-terminal extracellular region (ECR) and within the membrane-associated portion of PC1 are indicated along with identified functional roles. ECM, extracellular matrix; GPS, GPCR proteolysis site; GAIN, GPCR autoproteolysis inducing; TM, transmembrane, S, transmembrane segment; PLAT, polycystin/lipoxygenase/α toxin; TOP, Tetragonal Opening of Polycystins; LRR, leucine-rich repeats; CTL, C-type lectin; WSC, cell-wall integrity and stress-response component; FN, fibronectin-like. The region of PC1 with sequence and structural homology to the ion channel polycystin-2 (PC2) is indicated in green. **(B)** The cleavage products of PC1. Shown are the N-terminal fragment (NTF) and C-terminal fragment (CTF) that result from auto-catalyzed GPS cleavage and separation from each other. Separation of the NTF and CTF subunits exposes the stalk consisting of the final, 13th beta strand (red arrow) of the intact GAIN domain and a linker, which then constitutes the N-terminus of the CTF. Approximate locations of the protease-mediated cleavage sites within the CTF are indicated by the yellow arrows.

The *PKD1* gene was identified over a quarter of a century ago (The European Polycystic Kidney Disease Consortium, 1994; The International Polycystic Kidney Disease Consortium, 1995; Hughes et al., 1995). The PC1 protein sequence of 4,302 residues was proposed to have multiple membrane-spanning domains flanked by an extensive N-terminal extracellular region (ECR) and a much shorter cytosolic C-terminal tail (C-tail) (Figure 1A). These early sequence analyses suggested the possibility of 7–13 membrane-spanning domains, however it was not until the sequencing of the pufferfish *Pkd1* gene that the field began to settle on an 11-TM domain conformation (Sandford et al., 1997). Biochemical approaches utilizing N-linked glycosylation analyses subsequently confirmed the integral membrane status, topology, and 11-TM structure of PC1 (Boletta et al., 2001; Nims et al., 2003). A cryo-EM-based structure of the membrane-integrated portion of PC1 in complex with PC2 was solved in 2018 (Su et al., 2018). This work provided final proof of an 11-TM structural conformation for PC1 with a > 3,000 residue N-terminal region and a < 200 residue cytosolic C-tail. Importantly, the region of PC1 encompassed by the last 6 TM domains, which was originally noted to share homology with the sequence of PC2 (Mochizuki et al., 1996), was found to have an ion channel-like structure (Su et al., 2018) (Figure 1). Using the nomenclature adapted from ion channels, this region of PC1 consists of a voltage-sensing domain (S1-S4), a potential pore-forming unit (S5-S6), and a large extracellular loop between S1 and S2 named the Tetragonal Opening of Polycystins (TOP) domain (Figure 1A). Such observations are consistent with a proposed ion channel subunit function for PC1 (Hanaoka et al., 2000) (see more below).

The PC1 ECR consists of multiple functional domains (Figure 1). One unique domain whose structure resembles an Ig-fold and is repeated 16 consecutive times was subsequently named the PKD repeat (Sandford et al., 1997). Atomic force microscopy analyses with bacterially-expressed PKD repeats demonstrated their mechanical strength, which was altered by changes in the solvent or by including ADPKD missense mutations, consistent with a suggested role in mechano- or force-sensing (Forman et al., 2005; Qian et al., 2005; Ma et al., 2009; Ma et al., 2010). PKD repeats also have the ability to interact with each other and have been shown to mediate cell-cell interactions (Ibraghimov-Beskrovnaya et al., 2000; Streets et al., 2003). Other ECR domains with homology to leucine-rich repeats (LRRs), a C-type lectin domain (CTL), and a cell-wall integrity and stress-response component (WSC) suggest a role for PC1 in cell adhesion, which was supported by *in vitro* binding studies demonstrating interactions between the LRR and CTL domains and various purified components of the ECM (Weston et al., 2003). Recently, the CTL and WSC domains were reported to bind secreted Wnts (Kim et al., 2016) and the LRR domain was implicated in activation of the ion channel activity of PC2 (Ha et al., 2020). The membrane proximal portion of the PC1 ECR was noted to have homology with the sea urchin Receptor-for-Egg-Jelly (suREJ) protein involved in the sperm acrosome reaction (Moy et al., 1996) and was thereby called the REJ module (Sandford et al., 1997). This region was found to consist of a fibronectin-like fold and a unique structure called the GPCR autoproteolysis inducing (GAIN) domain, which undergoes autocatalytic proteolytic cleavage at a conserved GPCR proteolysis site (GPS) (Arac et al., 2012; Xu et al., 2013). GPS cleavage of PC1 creates an extracellular N-terminal fragment (NTF) and a membrane embedded C-terminal fragment (CTF) (Figure 1B), which remain non-covalently associated (Qian et al., 2002), and likely play important roles in PC1 function (see Section 3 for further details).

In addition to GPS cleavage, the membrane-associated portion of PC1 undergoes protease-mediated cleavage at multiple sites (Figure 1B). These sites are located within the loops between TM4-TM5, TM5-S1, S1-S2 (TOP domain) and in the last TM domain and C-tail (Chauvet et al., 2004; Woodward et al., 2010; Talbot et al., 2011; Lea et al., 2020). The C-terminal fragments produced from these cleavage events have been observed in either cell culture, kidney tissue, or urinary exosomes, and a variety of roles have been ascribed for some of them (e.g., as regulators of transcription, store-operated calcium entry, cytokine expression, and mitochondrial function) (Lal et al., 2008; Woodward et al., 2010; Talbot et al., 2011; Azevedo et al., 2018). Notably, the two C-tail cleavage fragments are able to undergo nuclear translocation *via* an intrinsic nuclear translocation signal or a transcription factor binding partner, respectively (Chauvet et al., 2004; Talbot et al., 2011).

Multiple functional roles have been described for domains or motifs located within the membrane-associated portion of PC1. The polycystin/lipoxygenase/α toxin (PLAT) domain, which comprises most of the first intracellular loop, regulates the membrane trafficking of PC1 by its ability to bind phosphatidylserine, PI_4_P, and β-arrestin (Xu et al., 2016). Binding of β-arrestin to 7-TM GPCRs is typically induced by GRK phosphorylation of the GPCR following its activation of heterotrimeric G proteins and can result in downregulation of G protein signaling or can promote β-arrestin-mediated signaling (Jiang et al., 2022). For PC1, β-arrestin-binding is regulated by phosphorylation at a nearby PKA site (S3164), and when bound by β-arrestin, PC1 is removed from the membrane (Xu et al., 2016).

A short sequence within the C-tail that was capable of stimulating GTPase activity of Gαi/o when tested as a synthetic peptide was named the G protein activation motif and led to a proposed GPCR-like function for PC1 ((Parnell et al., 1998); see Section 2 for more). Two different motifs involved in ciliary targeting of PC1 have been described within the C-tail: KVHPSST at the C-terminus (Ward et al., 2011) and a sequence that overlaps with the G protein activation motif and the binding sites for protein phosphatase 1 and calmodulin (Parnell et al., 2012; Doerr et al., 2016; Luo et al., 2019). Finally, the membrane-distal portion of the PC1 C-tail was discovered to contain a coiled-coil domain that interacts with the PC2 C-tail (Qian et al., 1997; Tsiokas et al., 1997) and other protein partners (Hardy and Tsiokas, 2020), most of which have roles that remain to be determined.

As one might expect from its structural complexity, a multitude of functions have been proposed for PC1. In addition to a role in cell adhesion based on its ECR domains, PC1 is reported to functionally interact with cadherins and to be localized to multiple plasma membrane domains, including adherens junctions, desmosomes, focal adhesions, and the primary cilium (Huan and van Adelsberg, 1999; Scheffers et al., 2000; Yoder et al., 2002). Interactions between PC1 and cytoskeletal elements have been described, as have PC1-dependent effects on cell polarity, cell migration, and planar cell polarity (Castelli et al., 2013; Yao et al., 2014; Castelli et al., 2015; Nigro et al., 2015). A number of early studies involving ectopic expression of various PC1 C-terminal expression constructs implicated a role in cellular signaling for PC1. These included an ability to activate signaling pathways to AP-1 (involving Cdc42, Rac-1, PKC, JNK, and heterotrimeric G proteins) (Arnould et al., 1998; Parnell et al., 2002), TCF (*via* β-catenin stabilization) (Kim et al., 1999a), and NFAT (*via* Gαq, PLC, and intracellular calcium) (Puri et al., 2004). An ability of the PC1 C-tail to bind and activate G proteins led to an early proposal that PC1 functions as an atypical GPCR (see Section 2). Later, full-length PC1 was shown to activate a p21 gene promoter (*via* JAK2/STAT1) in a PC2-dependent manner (Bhunia et al., 2002) and to regulate tubule versus cyst formation in 3D collagen gel assays. Roles for PC1 in the modulation of a variety of signaling pathways have been proposed, including Wnt signaling (Kim et al., 1999a), STAT regulation (Weimbs et al., 2013), and YAP/TAZ activity (Nigro et al., 2019), among others.

### 1.3 Polycystin-1 as an ion channel subunit

PC1 is thought to form a membrane receptor-ion channel complex with PC2 that is responsive to ligand- or mechanical-activation and that plays a role in cellular ion homeostasis. This concept is based on the homology between PC1 and the ion channel structure of PC2 and demonstration of PC1/PC2 interaction and complex formation (Newby et al., 2002). Studies in transfected CHO cells revealed unique, PC1 and PC2 co-dependent, calcium-permeable cation currents (Hanaoka et al., 2000). However, the idea of a joint ion channel function for these two proteins did not become widely recognized until the ability of PC1/PC2 to sense fluid shear stress and modulate intracellular calcium levels was demonstrated in cells (Nauli et al., 2003; Alenghat et al., 2004; Nauli et al., 2008; MacKay et al., 2022). Most recently, endothelial cell-specific *Pkd1* and *Pkd2* knockout mice were used to demonstrate regulation of vasodilation by the PC1/PC2 complex (MacKay et al., 2022). Formation of an ion channel-like structure consisting of PC1 and PC2 was finally validated by solving the molecular structure of the membrane-associated portions of the two proteins together (Su et al., 2018). The cryo-EM structure revealed a heterotetrameric complex consisting of three PC2 subunits and one PC1 subunit, as originally proposed (Yu et al., 2009; Zhu et al., 2011). Although the putative pore loops of the PC1 subunit were not visible, an ion conduction pore formed by the final two TM domains of each subunit was discernable. Electrophysiological studies in *Xenopus* oocytes recently demonstrated a direct role of the PC1 subunit in the ion channel activity of the PC1/PC2 complex (Wang et al., 2019). Co-expression of PC1 together with a gain of function (GOF) mutant of PC2 resulted in ion channel properties that differed from the homotetrameric PC2 GOF channel, including an increased permeability for Ca^2+^. Missense mutations within the putative pore region of PC1 resulted in significant alterations in ion permeability and current characteristics of the PC1/PC2 GOF complex demonstrating a direct role for PC1 in formation of the pore and thus in ion channel activity. This work also showed that GPS cleavage of the PC1 subunit was not required for channel activity and that complexes formed with the CTF or the six C-terminal TM domains of PC1 were also capable of ion conductance.

Relatively little is known regarding the regulation of this unusual PC1/PC2 ion channel complex. Binding of Wnts to the CTL and WSC domains of the PC1 ECR results in ligand-mediated activation of the PC1/PC2 ion channel complex (Kim et al., 2016). In an intriguing twist, the LRR domain within the PC1 ECR was shown to bind to N-glycans of the PC2 TOP domain and to activate the ion conductance of the complex (Ha et al., 2020). In this latter work, the PC1 NTF was proposed to act as a soluble ligand that activates the PC1/PC2 receptor-ion channel complex. Currently, there is only a single study suggesting that PC1-mediated G protein signaling regulates PC1/PC2 channel activity (Parnell et al., 2018). In contrast, an earlier study proposed that channel activation occurred *via* conformational rearrangements of PC1 (Delmas et al., 2004). As such, this is an important aspect of polycystin function that remains to be clarified by further investigation.

In summary, while cellular adhesion, signal transduction, and ion channel activity have all been identified as PC1 functions, how these functions are interconnected and which specific function whose loss initiates cystogenesis remains unresolved. The focus of this article is on the evidence that suggests a critical function of PC1 is to regulate heterotrimeric G protein signaling.

## 2 Evidence for polycystin-1 GPCR function

### 2.1 Polycystin-1 interacts with heterotrimeric G proteins

**FIGURE 2:**
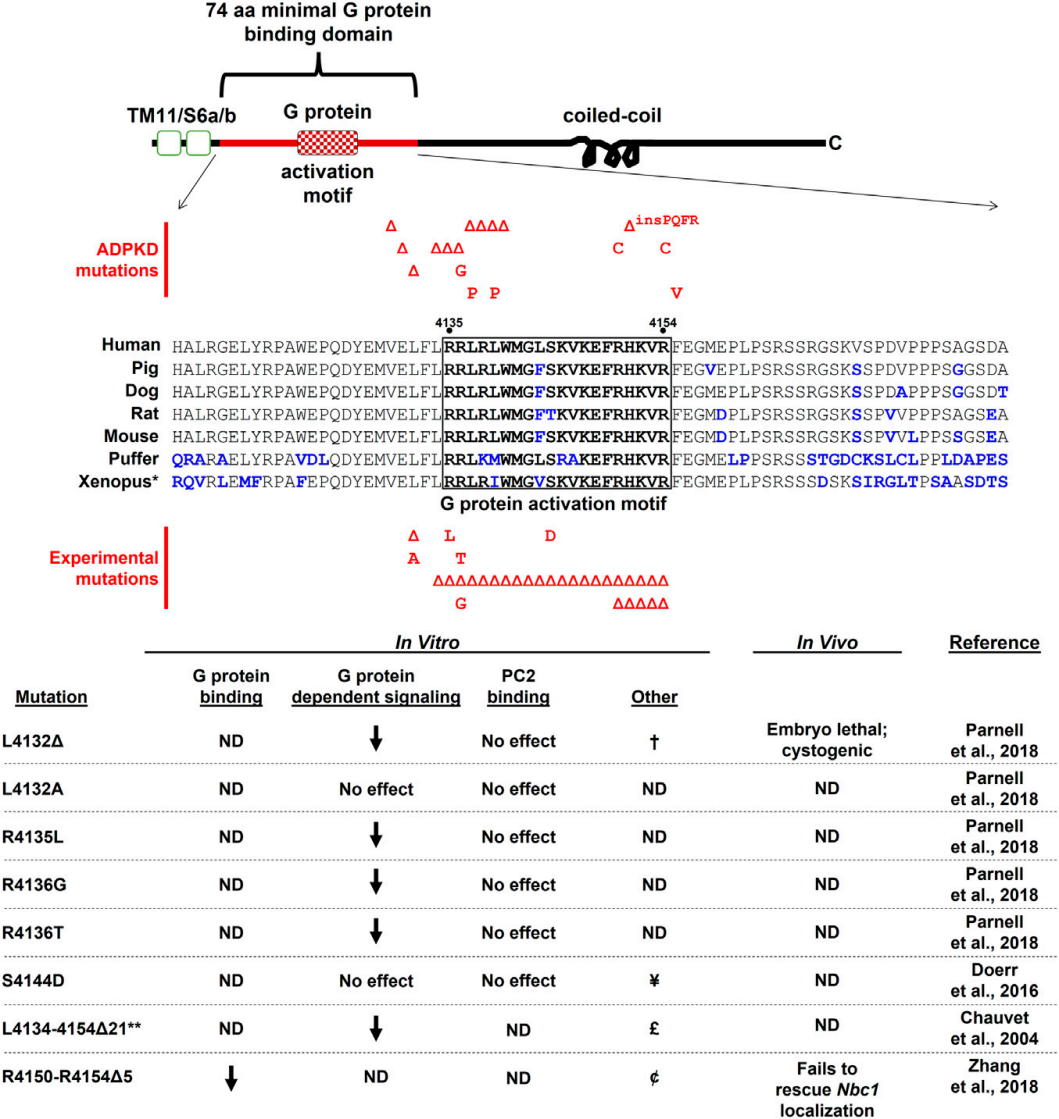
The cytosolic C-terminal tail of PC1 is shown schematically with sequence alignment of the 74 aa minimal G protein binding domain (corresponding to human aa 4,111–4,184). Small deletion and missense ADPKD-associated mutations that fall within this region and score as “Pathogenic” or “Likely Pathogenic” (as determined by the ADPKD Variant Database <pkdb.mayo.edu>) are shown above the sequence alignment. Experimentally-generated mutations designed to test effects on G protein signaling are shown below the alignment. Additional references for these ADPKD-associated mutations can be found at (Afzal et al., 1999; Perrichot et al., 1999; Garcia-Gonzalez et al., 2007; Rossetti et al., 2007; Reed et al., 2008; Tan et al., 2009; Audrezet et al., 2012; Rossetti et al., 2012). ND, not determined; †, decreased PC1/PC2 channel activity; ¥, calmodulin-binding disrupted, decreased PC1/PC2 channel activity and flow-dependent channel response, no effect on ciliary localization, decreased energy metabolism; £, decreased nuclear localization; ¢, C-tail nuclear localization unaltered. *Xenopus sequences are from *X. tropicalis*; **construct expressed as a soluble protein. Sequence accession numbers: human AAC37576; pig CBZ01637; dog AAM22956; rat AAG33986; mouse AAC53207; puffer XP_011610747; *Xenopus* XP_017952982.

The first evidence that PC1 could interact with heterotrimeric G proteins came from *in vitro* binding studies utilizing GST fusion proteins consisting of various portions of the C-tail of mouse PC1 (Parnell et al., 1998). Pull-down and co-immunoprecipitation assays demonstrated interactions between these C-terminal fusion proteins and heterotrimeric Gα and Gβ subunits from various sources, including heterotrimeric complexes purified from bovine brain and from rat brain lysates. These experiments also identified a membrane-proximal, minimal binding region of 74 amino acids required for stable interactions between PC1 and heterotrimeric G proteins that is highly conserved among vertebrates (Figure 1A, Figure 2). This minimal G protein binding domain contains a polybasic stretch of 20 amino acid residues that possess guanine-nucleotide exchange factor activity. Exchange factor activity was demonstrated by assays using purified heterotrimeric G proteins and a synthetic 20 amino acid peptide spanning this so-called G protein activation motif (Parnell et al., 1998). The minimal binding domain is distinct from the membrane-distal portion of PC1’s C-tail, which contains the coiled-coil domain responsible for interactions with PC2 (Qian et al., 1997) (Figure 1A, Figure 2). Several engineered mutations affecting PC1 function, as well as ADPKD-associated mutations, have been generated within and near the G protein activation motif (discussed further in sections 2.2, 2.3).

Additional studies have reported interactions between Gαi1, Gαi3, Gαs, and Gα12 using various approaches. Kwak et al. (2018) demonstrated an interaction between PC1 and Gαi3 by both co-immunoprecipitation and FRET between transiently-expressed constructs. Yuasa et al. (2004) found that, when expressed as a GST-fusion protein, the PC1 C-tail pulled down Gα12 subunits from transfected MDCK cell lysates, and endogenously expressed Gαs and Gαi1 subunits. Stable interactions were also detected between PC1 and Gα12^Q229L^ (Aragay et al., 1995), a mutant form of Gα12 that exists in a constitutively active state due to an inability to hydrolyze GTP. Yu et al. (2010) demonstrated stable interactions between PC1 and Gα12, and subsequently Yu et al. (2011) identified mutations within Gα12 that completely disrupted interactions with PC1. Importantly, this work also identified the previously characterized 74 amino acid minimal G protein binding domain within the C-tail of PC1 as essential for the PC1/Gα12 interaction. An endogenous interaction between PC1 and Gαi2 in mouse embryonic fibroblasts was also suggested by studies utilizing the *Pkd1* ^*HA/HA*^ mouse model and an approach involving SILAC coupled with immunoprecipitation and mass spectrometry (Nigro et al., 2019). Stable interactions between the C-tail of PC1 and various Gα protein subunits were also demonstrated by surface plasmon resonance in a screen of bacterially-expressed PC1 C-tail constructs (from *Xenopus*) and all Gα subunits found in the *Xenopus* embryonic pronephros expressed in reticulocyte lysates. High-affinity interactions were detected both ways between the C-tail and Gnas, Gna14, Gnai1, and Gnai2, as well as mouse Gna12 (Zhang et al., 2018). These binding affinities were comparable to those found for Gα subunits and other GPCRs (Komolov et al., 2006), and binding was completely disrupted by deletion of 5 amino acids from the previously identified G protein activation motif (Figure 2).

The minimal binding domain for G proteins also overlaps with other previously described regions of interest, including binding sites for calmodulin (Doerr et al., 2016) and protein phosphatase 1 (Parnell et al., 2012), a protein kinase A phosphorylation site (Parnell et al., 1999), and sequences for ciliary (Luo et al., 2019), mitochondrial (Lin et al., 2018), and nuclear (Chauvet et al., 2004) localization, suggesting that G proteins may be involved in multiple PC1 functions. The distal portion of the C-terminal tail of PC1, beyond the minimal G protein binding domain, has also been shown to interact with RGS7 (Kim et al., 1999b). RGS7 is a member of the negative Regulator of G protein Signaling family capable of stimulating the GTPase activity of Gα subunits, resulting in their inactivation (Dohlman and Thorner, 1997). This interaction was identified genetically by a yeast two-hybrid screen and physically *via in vitro* binding assays and co-immunoprecipitation of transiently-expressed components. Co-expression of the C-tail of PC1 altered the cellular localization of RGS7 and prevented its degradation in transfected cells, further suggesting a physical interaction between the two proteins.

### 2.2 Polycystin-1 regulates heterotrimeric G protein signaling

In addition to direct interactions between PC1 and heterotrimeric G protein subunits, numerous lines of evidence have suggested that PC1 regulates heterotrimeric G protein-dependent signaling in cellular assay systems. In MDCK cells ectopically expressing PC1, resistance to apoptosis was shown to be dependent on PI3Kβ activation *via* heterotrimeric G proteins, by using pertussis toxin (Boca et al., 2006). PC1-mediated activation of c-Jun-N-terminal kinase (JNK) and AP-1 promoter-reporter activity was inhibited by Gβγ-sequestering βARK-ct, dominant-negative Gαi2, and the Gα12/13 dominant-negative inhibitor p115RhoGEF (Parnell et al., 2002). In this study, PC1-dependent JNK and AP-1 activity was augmented by co-transfection of WT Gα subunits, including Gαi1, Gαi2, Gαi3, Gα12/13, and Gαq. Gαq also potentiated activation of PC1-dependent NFAT promoter-reporter activity (Puri et al., 2004). Mutation of critical amino acids within the G protein activation motif, including ADPKD patient-associated mutations [see Figure 2 and (Afzal et al., 1999; Perrichot et al., 1999; Garcia-Gonzalez et al., 2007; Rossetti et al., 2007; Reed et al., 2008; Tan et al., 2009; Audrezet et al., 2012; Rossetti et al., 2012)], also reduced PC1-mediated basal and G protein-augmented activation of AP-1 promoter reporter activity (Parnell et al., 2018). Of note, a single amino acid ADPKD patient mutation L4132Δ (Afzal et al., 1999) was found to block basal and augmented AP-1 activity as well as PC1/PC2 channel activity in electrophysiological studies in CHO cells (Parnell et al., 2018). This mutation is one of several ADPKD-associated small deletion mutations (see Figure 2), interspersed over amino acids 4,130–4,140, that are predicted to disrupt the amphipathic helical structure of the 20 amino acid G protein activation motif. Within this cluster of mutations are two proline substitutions that are likely to break the localized helical structure of the motif. Notably, an experimentally engineered substitution L4132A, which would presumably allow retention of the amphipathic nature of the activation motif, did not block basal AP-1 activation. Deletion of the entire G protein activation motif in the context of a soluble C-terminal tail fragment of PC1 blocked both AP-1 activity and nuclear translocation of the soluble fragment (Chauvet et al., 2004). These results suggest that PC1 regulates cellular signaling pathways, including PC2 channel activity, by activating heterotrimeric G protein signaling.

In additional electrophysiological studies, expression of PC1 in sympathetic neurons that do not otherwise express PC1 resulted in modulation of Ca^2+^- and GIRK-channel activity. PC1-mediated channel modulation could be prevented by inhibitors of G protein signaling, including Gβγ-sequestering Gα transducin, non-hydrolyzable GDP-β-S, and pre-treatment of cells with pertussis toxin and N-ethylmaleimide. In this assay system, G protein-dependent channel regulation was antagonized by co-assembly of PC1 with co-expressed PC2 (Delmas et al., 2002). A later study by this same group showed that structural rearrangement of PC1 simultaneously but independently stimulated the channel activity of PC2 itself and G protein-dependent signaling (Delmas et al., 2004). These results suggest that G protein-dependent signaling and channel activity of the PC1/PC2 complex are coordinately regulated, potentially *via* a ligand-mediated structural rearrangement of PC1.

Co-transfection of PC1 and PC2 with activator of G protein signaling 3 (Ags3) increased PC1/PC2 channel activity, and this activity could be inhibited by co-transfection of Gβγ-sequestering βARK-ct (Kwon et al., 2012). Co-transfection of PC1 with TRPC4β increased TRPC4β-dependent channel currents and increased the amount of Gαi3 in complex with TRPC4β. These currents were inhibited by a dominant-negative Gαi3 (Kwak et al., 2018). These results suggest that PC1-mediated activation of heterotrimeric G protein signaling can regulate the activity of numerous channel proteins. Interestingly, GPS-cleavage-deficient PC1 mutants were incapable of TRPC4β activation (Kwak et al., 2018), suggesting a potential link between GPS cleavage and G protein activation (see Section 3).

**FIGURE 3:**
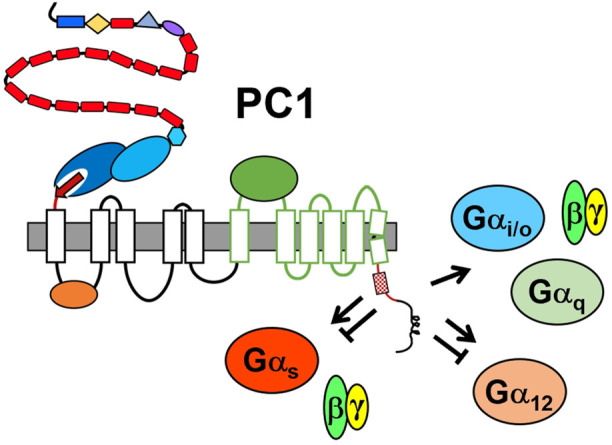
Summary of PC1-mediated regulation of heterotrimeric G protein signaling. The minimal G protein binding domain is represented by the membrane-proximal portion of the C-tail, i.e., red line with hatched box representing the G protein activation motif. Other structural domains are as identified in Figure 1. Arrow indicates activation; the bar-headed line indicates inhibition. There is evidence that PC1 both positively and negatively regulates Gαs and Gα12 families.

Several lines of evidence have also suggested that PC1 negatively regulates G protein signaling. Activation of Gα12 in MDCK cells induces JNK and stimulates apoptosis. This Gα12-stimulated activity is enhanced by silencing of PC1 and is inhibited by over-expression of PC1 (Yu et al., 2010). PC1-dependent inhibition of Gα12-mediated apoptosis is abrogated by deletion of the minimal G protein binding domain and by Gα12 mutations that uncouple binding between Gα12 and PC1 (Yu et al., 2010; Yu et al., 2011). PC1 silencing or Gα12 activation also promoted increased shedding of E-cadherin and nuclear localization of β-catenin in an ADAM10-dependent fashion (Xu et al., 2015), and altered expression of N-cadherin from early-to late-isoforms in MDCK cells (Wu et al., 2016). In an assay of mouse proximal tubular cells grown in matrigel, loss of PC1 resulted in cyst formation, but treatment of these cells with a small-molecule inhibitor of Gβγ subunits, gallein, inhibited cell proliferation and promoted tubule formation (Zhang et al., 2018). Thus, PC1 appears to have the potential to both positively and negatively regulate heterotrimeric G protein signaling (see Figure 3).

### 2.3 Evidence from animal models

Several of the studies offering cell-based evidence for PC1-mediated regulation of G protein signaling are complemented by experiments performed in animal models. In Parnell et al. (2018) the ADPKD patient mutation L4132Δ was introduced into the mouse *Pkd1* gene. This single amino acid deletion mutation, which blocked PC1/G protein mediated activation of promoter-reporter activity as well as PC1/PC2 channel activity in cellular assays, also resulted in a severe loss of PC1 function as evidenced by a cystic embryonic kidney phenotype and embryonic lethality in *Pkd1* ^Δ*L/*Δ*L*^ embryos, and rapid cyst formation in newborn *Pkd1* ^Δ*L/*fl^ following Hoxb7 Cre-mediated excision of the floxed allele. These results suggest that the ΔL mutation, thought to interfere with the local structure of the C-tail G protein activation motif, prevents an essential function of the PC1 protein, namely G protein activation. In other experiments, Kwon et al. (2012) determined the consequence of Ags3 knockout in the context of the hypomorphic *Pkd1* ^*V/V*^ mouse. In cell-based assays, PC1/PC2 channel activity was increased by co-transfection of Ags3 in a Gβγ-dependent manner. Likewise, cystic disease in the *Pkd1* ^*V/V*^ mouse was exacerbated by homozygous deletion of Ags3. In contrast, however, Yu et al. demonstrated that various Gα12-dependent signaling outputs, including JNK and apoptosis, were upregulated in the absence of PC1 and downregulated by its over-expression (Yu et al., 2010; Yu et al., 2011). Wu et al. (2016) subsequently demonstrated that genetic deletion of Gα12 completely blocked renal cystogenesis in *Pkd1* ^*fl/fl*^ mice with Mx1 Cre-driven deletion of *Pkd1*, suggesting that Gα12 is required for the development of renal cysts following loss of PC1 function. Finally, Zhang et al. (2018) demonstrated that treatment of *Xenopus* embryos with the Gβγ subunit inhibitor gallein, which inhibited cell proliferation and cyst formation in *Pkd1*-deficient proximal tubular cells, also prevented cystic phenotypes in *Xenopus Pkd1* morphants. A cystic phenotype could also be induced in *Xenopus* by morpholinos directed against cAMP-activating *Gnas*, and cystic *Xenopus* phenotypes were rescued by deletion of a Gβ subunit or by expression of the PC1 C-tail. However, a C-tail construct with a mutation within the G protein binding domain was not capable of rescuing the *Pkd1* morphant phenotype. Refer to Figure 2 for a summary of the effects of mutations within the minimal G protein binding domain observed in *in vitro* and *in vivo* experimental systems.

While these various cellular and *in vivo*-based experiments describe potential links between PC1 and G protein dependent signaling, it is important to note that a limitation of these studies is that they do not distinguish between effects that are dependent on a direct interaction between PC1 and heterotrimeric G proteins versus G protein-dependent effects that are downstream of PC1-dependent signaling. Additional experimentation will likely be required to resolve these questions.

## 3 Polycystin-1 as a novel adhesion GPCR

### 3.1 Adhesion G protein-coupled receptors, G protein-coupled receptors proteolysis site cleavage and the GAIN domain

One of the defining and generally conserved structural features of the PC1 protein family identified early on (Ponting et al., 1999) is the presence of a GPCR proteolysis site, or GPS, that is now known to be part of a larger, evolutionarily conserved structure named the GAIN domain (Arac et al., 2012). The GAIN domain and its unusual properties (see below) are found only in the PC1 and adhesion GPCR families (Promel et al., 2013). Adhesion GPCRs play important functions in planar cell polarity, neuronal development, and tumor cell biology among others (Maser and Calvet, 2020; Lala and Hall, 2022). Unlike other GPCR families they typically have extremely large, extracellular N-terminal regions. Furthermore, the ECRs of adhesion GPCR proteins are composed of multiple types of “adhesive” domains (e.g., LRR, Ig-like, lectin) that are often involved in cell-cell and cell-matrix interactions. Together with the GAIN domain, these ECR properties and functions represent additional features shared between adhesion GPCRs and PC1.

The GAIN domain and GPS motif were named due to their involvement in a proteolytic reaction that occurs at a conserved [HL**↓** ^T^/_S_] tripeptide (where **↓** indicates the cleavage site) within the GPS motif. The GPS motif is an ∼50 residue sequence characterized by conserved tryptophan and 2-4 disulfide bond-forming cysteine residues. This motif is located in the extracellular N-terminal region of both adhesion GPCRs and PC1 in close proximity to the first TM domain and is part of a larger (∼300 residue) GAIN domain (Arac et al., 2012). The prototypical GAIN domain consists of two subdomains, A and B, and is composed of 8 alpha helices and 13 beta strands, in which strands 9–13 make up the GPS motif. Cleavage at the GPS occurs *via* an autocatalytic cis-proteolytic reaction facilitated by nucleophilic residues surrounding the cleavage site. GPS cleavage generates a C-terminal, membrane-embedded fragment, the CTF, and an N-terminal, extracellular fragment, the NTF. The NTF and CTF subunits remain non-covalently attached through numerous hydrophobic and H-bond interactions between the final 13th beta strand and strands 6, 7 and 9 within the C-terminal B subdomain. Since this seminal discovery, the GAIN domain structures of GPR56/ADGRG1 and GPR126/ADGRG6 (Salzman et al., 2016; Leon et al., 2020) have also been solved and revealed only slight variations, primarily in size, in the overall composition and structure of this domain.

### 3.2 Mechanisms of G protein activation by adhesion GPCRs

As befits their complex structural organization, the regulation of G protein signaling by individual adhesion GPCRs has been shown to involve multiple mechanisms. Several groups reported that expression constructs encoding the CTF subunit alone, beginning with the first residue following GPS cleavage, were capable of activating heterotrimeric G proteins in a constitutive manner [reviewed in (Maser and Calvet, 2020)]. When compared to the NTF/CTF heterodimer, CTF-mediated activation was much greater for a number of adhesion GPCRs. These observations were originally interpreted to suggest that the associated NTF subunit had a role in inhibiting signaling by the CTF. Thereafter, two different groups showed that the constitutive signaling activity of the CTF subunit was dependent on the presence of the short, N-terminal ‘stalk’ preceding the first TM domain (Liebscher et al., 2014; Stoveken et al., 2015). This requirement for the stalk for activation of adhesion GPCRs was demonstrated by the inability of CTF constructs with deletion of the stalk to signal. The ability of soluble, synthetic peptides derived from the stalk sequence to rescue G protein signaling by the stalk-deleted mutants provided additional support for the stalk-dependent mechanism. To fit these and preceding observations, the ‘tethered cryptic ligand/agonist’ model for activation of G protein signaling by adhesion GPCRs was proposed (Liebscher et al., 2014; Stoveken et al., 2015).

In the tethered cryptic agonist model, GPS cleavage followed by dissociation of the NTF results in exposure of the stalk/tethered agonist (TA) previously buried within the GAIN domain. Exposure (de-cryption) of the largely hydrophobic stalk was proposed to favor its subsequent interaction with the membrane-embedded 7-TM helical bundle of the CTF, presumably leading to conformational changes which would drive heterotrimeric G protein binding and activation. Since proposal of this mechanism, the CTF stalk has also been referred to as the tethered peptide ligand, TA, or Stachel sequence (Stachel being German for *stinger*). Proponents of the TA model envisioned that the NTF might be removed from the CTF subunit *via* mechanical means or its interactions with an adhesion ligand.

Activation of signaling by the CTF as a direct consequence of NTF dissociation has since been demonstrated by replacing the GAIN domain and the GPS cleavage site with the recognition site for an exogenous protease such as thrombin or enterokinase. Protease treatment of cells expressing these chimeric adhesion GPCRs was shown to result in the exposure of the TA and led to activation of signaling (Mathiasen et al., 2020; Frenster et al., 2021; Lizano et al., 2021). Interaction with ECM binding partners followed by activation of signaling has also been demonstrated for a number of adhesion GPCRs, as has the application of mechanical stimulation by vibration, shaking, or shear stress (Petersen et al., 2015; Wilde et al., 2016; Yeung et al., 2020). Studies in *Drosophila* have shown a role for the adhesion GPCR latrophilin/dCIRL in mechanosensing by chordotonal neurons that involves regulation of TRP channel activity (Scholz et al., 2015; Scholz et al., 2017). Furthermore, there are links between missense mutations in EMR2/ADGRE2 and defects in VLGR1/ADGRV1 with familial vibratory urticaria and hearing loss, respectively (McMillan and White, 2010; Naranjo et al., 2020; Kusuluri et al., 2021). Such observations support a general view that the structural conformation of adhesion GPCRs is especially conducive to mechano-responsive signaling functions [reviewed in (Lin et al., 2022)].

Following proposal of the cryptic TA mechanism, its general applicability was challenged by observations of non-cleavable and heterodimeric, NTF/CTF-associated adhesion GPCRs that were still capable of signaling (e.g., GPR114/ADGRG5) (Wilde et al., 2016). Interestingly, for some cleavage-defective adhesion GPCRs, G protein signaling remained dependent on the TA (Bohnekamp and Schoneberg, 2011; Promel et al., 2012; Wilde et al., 2016; Scholz et al., 2017). A unifying paradigm for these observations was recently provided by experiments that utilized biorthogonal click-labeling to identify solvent-exposed TA residues (Beliu et al., 2021). Together with molecular dynamics simulations, this study revealed an inherent conformational flexibility within the GAIN domain. Two flexible loops or flaps were identified in the GAIN domain which appear to open and thereby allow portions of the TA sequence to become accessible for interaction with the TM bundle. It was postulated that ‘flexing’ of the GAIN domain might be modulated by the engagement of specific ligands to the GAIN domain itself (e.g., synaptamide), or to an adhesion domain within the ECR. Some groups have used the binding of synthetic ligands, such as antibodies directed at ECR domains or at ectopic N-terminal epitope tags, as a means to activate adhesion GPCRs (Salzman et al., 2017; Bhudia et al., 2020; Huang et al., 2020; Mitgau et al., 2022). Recently, the cryo-electron micrograph (EM) structures of the CTF subunit for a number of adhesion GPCRs were published by four groups (Barros-Alvarez et al., 2022; Ping et al., 2022; Qu et al., 2022; Xiao et al., 2022). These structures confirmed the originally proposed interaction of the TA in a hydrophobic binding pocket formed by the 7-TM helical bundle. Furthermore, the cryo-EM structure of a full-length, GPS cleavage-defective adhesion GPCR, GPR110/ADGRF1, revealed that its TA was able to bind in the 7-TM pocket (Qu et al., 2022). This structural evidence demonstrates the flexibility of the GAIN domain and supports this mechanism as another means of adhesion GPCR activation by its TA.

### 3.3 GPS cleavage is critical for Polycystin-1 function and prevention of cystogenesis

PC1 GPS cleavage occurs both *in vitro* and *in vivo* (Qian et al., 2002; Yu et al., 2007) to yield ∼300 kDal NTF and ∼130 kDal CTF subunits (Figure 1B), which can be found non-covalently associated or as separate subunits. GPS cleavage is ubiquitous, but incomplete, as shown by the presence of both full-length uncleaved and cleaved NTF/CTF forms of PC1 in multiple tissues and cell lines (Yu et al., 2007; Castelli et al., 2013; Kurbegovic et al., 2014). The relative proportion of cleaved versus uncleaved PC1 isoforms varies between tissue types and at different developmental stages (Castelli et al., 2013; Kurbegovic et al., 2014). Such observations suggest that this autocatalytic event can be regulated by additional factors, perhaps *via* conformational changes of the GAIN domain induced by the binding of ligands to various domains of the ECR. In support of this idea, the ER-resident protein, Sec63, involved in translocation of integral membrane and secreted proteins, has been implicated as being necessary for GPS cleavage of PC1 (Fedeles et al., 2015), as has the presence of PC2 (Chapin et al., 2010; Gainullin et al., 2015). It is also possible that the non-cleaved, NTF/CTF-associated or -dissociated isoforms of PC1 carry out distinct functions.

Approximately 30% of the missense mutations identified in *PKD1* are located within the GAIN domain or near the GPS motif. Studies in cultured cells have shown that many of these mutations reduce or prevent GPS cleavage and inhibit the ability of PC1 to both activate certain signaling pathways and induce tubulogenesis of MDCK cells in 3D collagen gels (Qian et al., 2002; Arac et al., 2012; Qian and Li, 2015). GPS cleavage may also be necessary for the proper maturation and trafficking of PC1 to the primary cilium (Cai et al., 2014; Kim et al., 2014; Su et al., 2015). Two *Pkd1* mouse models, each with defective GPS cleavage of PC1 due to a different missense mutation, have demonstrated that cleavage is essential for preventing cyst formation (Yu et al., 2007; Cai et al., 2014). Despite the likely importance of PC1 GPS cleavage, the mechanisms affecting cleavage and the role that GPS cleavage isoforms play in various PC1 functions, including the modulation of G protein signaling (Kwak et al., 2018), remain relatively unknown.

### 3.4 The adhesion GPCR-like signaling activation mechanism for polycystin-1

Although PC1 has been referred to as a novel or atypical adhesion GPCR based on the GAIN domain and GPS cleavage, TA-dependent signaling had not been reported until recently. In work from Pawnikar et al. (2022), transient transfection of wild-type (WT) or stalk-mutant CTF expression constructs of PC1 revealed a requirement for the stalk/TA in the activation of a promoter-luciferase signaling reporter. The stalk-mutant constructs included a CTF lacking the first 21 residues of the stalk (∆stalkCTF) and three additional proteins each with an ADPKD-associated missense mutation within the stalk- G3052R, R3063C and R3063P (ADPKD Variant Database, https://pkdb.mayo.edu). The CTF stalk mutants G3052R, R3063C, and ∆stalk displayed significantly reduced reporter activity in comparison to WT CTF. In another study, the CTF form of PC1 was shown to activate the NFAT reporter to a much greater extent than full-length PC1, and synthetic peptides derived from the CTF stalk sequence were able to stimulate NFAT reporter activation by ∆stalkCTF (Magenheimer et al., 2021). Work by Kwak et al. (2018) has shown that GPS cleavage of PC1 is required for activation of TRPC4 *via* Gαi3 in endothelial cells. Altogether, such observations are consistent with the PC1 CTF stalk possessing a TA-like activity that can mediate signaling by PC1.

**FIGURE 4:**
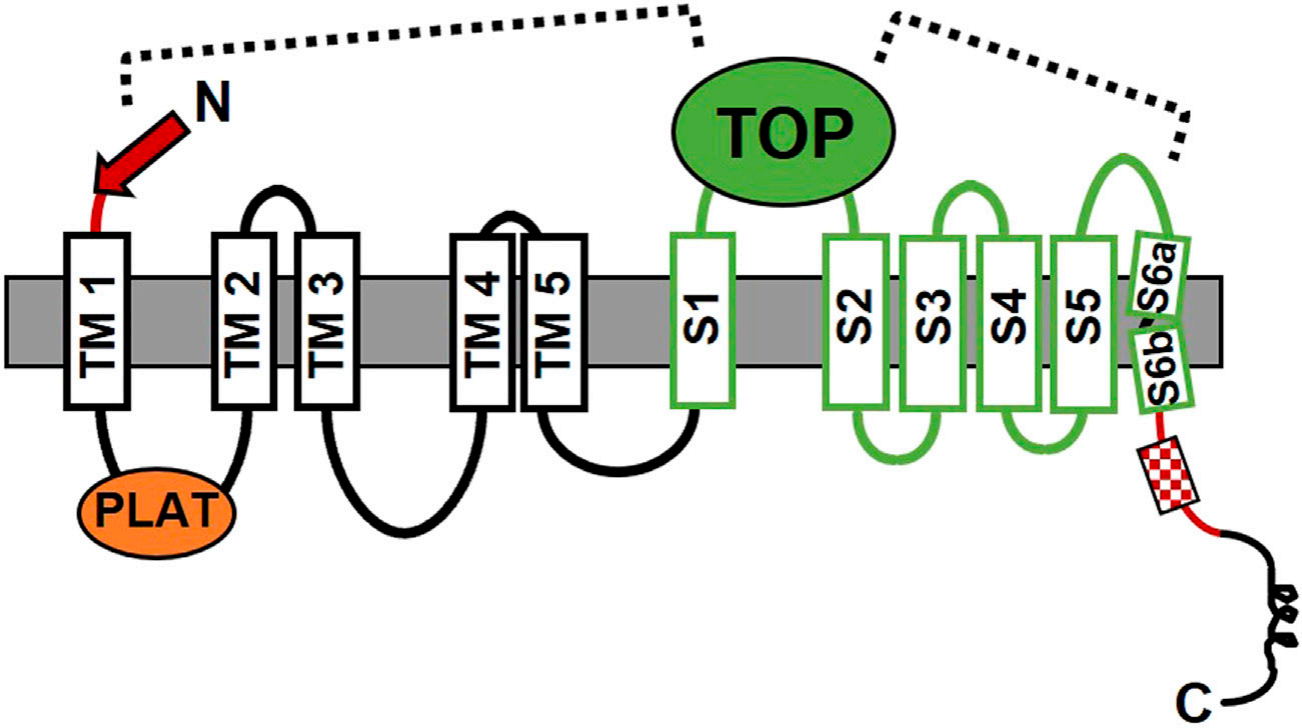
The proposed allosteric mechanism for stalk-mediated activation of signaling by the CTF form of PC1. The model is supported by the results from molecular dynamics simulations and mutagenesis-signaling studies (Pawnikar et al., 2022). The dashed lines indicate regions of the PC1 CTF protein where important correlated residue motions or residue-residue interactions were identified as being involved in activation of signaling by the stalk, i.e., between the N-terminal stalk (red arrow) and TOP domain, and between the TOP domain and the putative pore loop between S5 and S6 TM domains. Domains are as described in Figure 1; Section 1.

A potential mechanism for the stalk TA-mediated activation of the PC1 CTF was uncovered by molecular dynamics simulations using computer models of the WT and stalk-mutant CTF proteins, ∆stalkCTF, G3052R, R3063C and R3063P (Pawnikar et al., 2022). Highly correlated residue motions between the stalk-TOP and TOP-pore loop domains were observed for WT CTF (Figure 4) that were significantly lower in the stalk mutants, suggesting these domains were important for stalk TA-mediated signaling. Key residue-residue interactions between these regions were identified for WT CTF that appeared to be absent in simulations with the stalk mutants. Low-energy conformational states differed between WT and stalk-mutant CTF proteins and revealed that most of the key residue interactions identified in WT CTF were broken or absent in the stalk mutants. The importance of these residue-residue interactions was corroborated in functional cell signaling assays in which NFAT reporter activation was decreased for CTF expression constructs with single residue substitutions designed to disrupt key interactions. Such results are consistent with the proposal that an allosteric transduction pathway connecting the stalk-TOP-pore loop domains was responsible for stalk TA-mediated activation of signaling by the PC1 CTF. While consistent results were obtained in the studies described above, it is important to point out that both approaches involved examination of PC1 CTF alone, i.e., not in complex with PC2. The cryo-EM structure of PC1/PC2 complex revealed inter-subunit interactions between the TOP domains and the TOP domain with extracellular loops (Su et al., 2018). As such, the molecular mechanism for activation of signaling by the PC1/PC2 complex may differ from that of CTF alone.

It is tempting to speculate, based on the shared similarities between the ECRs of PC1 and adhesion GPCRs, that stalk TA-mediated activation of signaling by PC1 could be stimulated by its cell adhesion or mechanosensing properties. For example, components of the ECM interacting with the LRR, C-type lectin, or WSC domains of PC1 could serve as activating ligands. Similarly, trans-cellular (or even cis-cellular) interactions between the PKD repeats within the ECR of separate PC1 molecules, or shear stress might also serve as stimulatory signals (Ibraghimov-Beskrovnaya et al., 2000). In this scenario, these processes would remove the NTF, or alter the conformation of the GAIN domain, leading to exposure of the stalk TA of PC1. Once exposed, interaction of the stalk with the TOP domain would result in a signaling-active conformation of the CTF. So far, only Wnts and fluid shear stress have been identified as activating factors for PC1 using ion channel activity of the PC1/PC2 complex as a functional readout (Nauli et al., 2003; Kim et al., 2016). While the involvement of G protein signaling was ruled out in the case of shear stress (Nauli et al., 2003; Kim et al., 2016), the ability of Wnt9b binding to activate GPCR signaling by PC1 has been suggested in other work (Gresko et al., 2019).

Notably, the mechanism proposed for stalk TA-activated signaling by PC1 differs substantially from that recently revealed by the cryo-EM studies of adhesion GPCRs (Barros-Alvarez et al., 2022; Ping et al., 2022; Qu et al., 2022; Xiao et al., 2022). Whereas the PC1 CTF may be activated by interaction of the stalk TA with the extracellular TOP domain, for the adhesion GPCRs, the N-terminal end of the TA is inserted within the TM helical bundle. This disparity could be due to differences in their stalk/TA sequences and/or in the size and structure (and additional functions) of their extracellular loops. Furthermore, it is likely that the mechanism of TM signal transmission will also differ given the 11-TM versus 7-TM structural conformations of PC1 and adhesion GPCRs. Much work remains to be done to reveal the means of stalk TA-mediated TM signal transduction, and G protein selection and binding for PC1 (discussed further in Section 4), and it is anticipated that such insights will be novel and beneficial for our understanding of both atypical and canonical GPCRs.

## 4 Controversies regarding polycystin-1 G protein-coupled receptor function

A primary controversy that remains is the mechanism by which PC1 affects G protein function. Wu et al. demonstrated that *Pkd1* knockout led to increased Gα12 activation, and that genetic deletion of Gna12 in mice blocked cystogenesis induced by conditional deletion of *Pkd1* (Wu et al., 2016). In this model, PC1 is hypothesized to sequester Gα12 subunits that are putatively pro-cystogenic. In another study, Zhang et al. (2018) demonstrated a PKD phenotype in the pronephric Xenopus kidney following loss of Gαs, and that inhibition of Gβγ signaling antagonized this phenotype, suggesting that loss of Gαs leads to unregulated, cystogenic Gβγ signaling. This result suggests that PC1 inhibits PKD phenotypes in *Xenopus* by binding and sequestering Gαs in the heterotrimeric complex. These studies are in contrast with Parnell et al. (1998) who showed that PC1 contains a motif that activates guanine nucleotide exchange, and that a PC1 ADPKD patient mutation that disrupts G protein-dependent signaling in cellular assays results in severe cystic disease when introduced into a mouse model (Parnell et al., 2018). However, while these results may seem discordant, there are several potential explanations that may be able to reconcile these different models of PC1 function.

For one, PC1 appears to be promiscuous in its ability to interact with heterotrimeric G proteins, and may have differential effects on the activity of the various families or be affected by the context in which they interact. Paradigms for this model include the β_2_-adreneric receptor, which can activate either Gαs or Gαi, depending on the PKA phosphorylation status of the receptor (Lefkowitz et al., 2002), or the vasopressin V_2_ receptor, which has been shown to activate Gαs-dependent signaling and to inhibit Gα12 signaling in response to ligand-mediated activation (Okashah et al., 2020). In a similar manner, PC1 may activate a subset of G proteins under certain circumstances while binding and sequestering another subset of G proteins under other circumstances. It is also important to note that NAAIRS-based substitution of Gα12 did not identify its helix 5, which typically comprises ∼70% of the interaction surface between Gα subunits and GPCRs (Inoue et al., 2019), as a PC1 binding determinant. Instead, this analysis identified PC1-binding determinants in regions unique to Gα12, suggesting that different binding and regulatory properties may exist between PC1 and specific Gα families (Yu et al., 2011). Given this potential for both positive and negative regulatory mechanisms of interaction between PC1 and diverse Gα family members (Figure 3), it will be essential to determine the effects of knocking out other Gα family members on cystic disease initiation and progression.

Regardless of the mechanism by which PC1 regulates G protein signaling, it is important to note that all studies that describe any sort of interaction between PC1 and heterotrimeric G proteins ascribe central importance to the minimal G protein binding domain originally identified by Parnell et al. (1998). Given the centrality of this domain it is also essential to consider whether its presence (or absence) in model systems may affect experimental outcomes and interpretations. Ablation of Gα12 was seen to antagonize cystic disease in a mouse model with complete loss of the PC1 C-tail and G protein binding domain. However, perturbation of G protein signaling in other cystic models with intact C-terminal PC1 tails may yield different results than those observed in *Pkd1* conditional models that do not express any PC1. For instance, the hypomorphic *Pkd1* ^*RC*^ model is cystic due to decreased expression levels of PC1 (Hopp et al., 2012), but the protein retains an intact C-tail presumably capable of interacting with heterotrimeric G proteins and regulating their signaling properties *via* activation and/or sequestration. Likewise, cleavage mutants such as *Pkd1* ^*T3041V*^ (Yu et al., 2007) or signaling mutants such as *Pkd1* ^*ΔL*^ (Parnell et al., 2018) may potentially retain the ability to bind, but not signal to heterotrimeric G proteins. This line of thinking also begs the question of whether the expression levels of PC1 determine the mechanism by which it regulates different families of heterotrimeric G proteins. This question is particularly relevant given current interest in therapeutic approaches to ADPKD that involve re-expression of PC1 (Dong et al., 2021) or increasing *PKD1* and *PKD2* protein levels by blocking miR-17 (Lee et al., 2019; Lakhia et al., 2022). Since G protein activation is a catalytic event it would not require a large number of PC1 molecules to initiate signaling *via* activating mechanisms. In contrast, regulation of signaling *via* sequestration would be limited by the number of PC1 molecules available to interact. Thus, a thorough analysis of the role of G protein signaling in PC1 function may require the testing of a broader spectrum of PC1 mutants.

## 5 Time for a new paradigm

**FIGURE 5:**
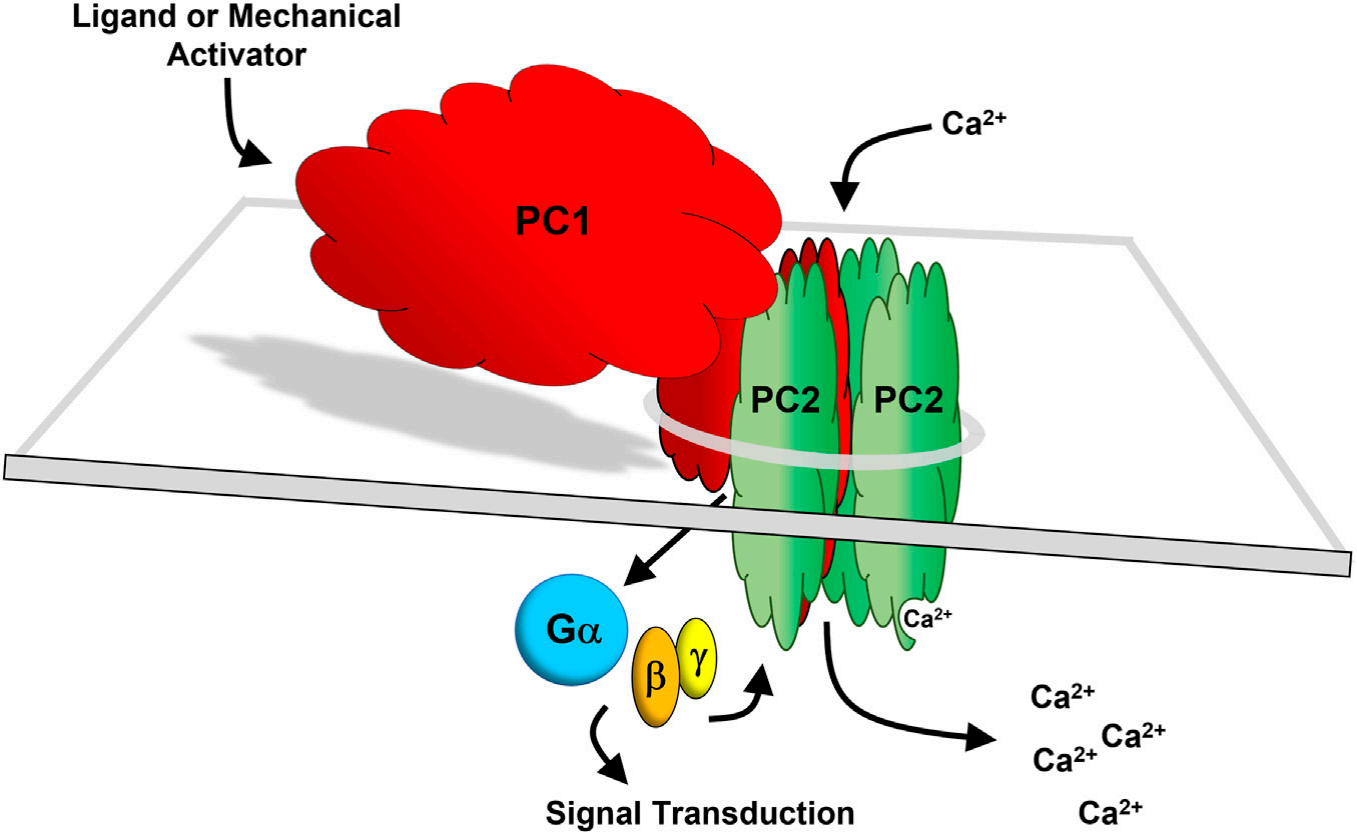
The PC1/PC2 hybrid receptor-channel complex. PC1 (red) and PC2 (green) are depicted as forming a heteromeric four-subunit complex comprised of three subunits of PC2 and one subunit of PC1 (Yu et al., 2009; Zhu et al., 2011; Wang et al., 2019). The last 6 TM domains of PC1 have homology with PC2. In this model, it is envisioned that PC2, being a transient receptor potential (TRP) channel (TRPP2) conducts a cation current together with PC1 acting as the fourth subunit of the channel. PC1 may function as a ligand-activated or mechanosensitive ionotropic receptor which transduces a signal to the channel subunits *via* heterotrimeric G protein activation (Parnell et al., 2018). Additionally, PC1 functioning as a G protein-dependent metabotropic receptor may independently activate downstream signal transduction.

As described in previous sections, PC1 appears to function as a ligand-activated and/or mechanosensitive adhesion GPCR and an ion channel subunit that forms a heterotetrameric channel with PC2. GPCRs are inherently metabotropic since they work through second messenger signaling mechanisms. In contrast, ionotropic receptors gate ions upon receptor activation. Based on current evidence, it would appear that PC1 may possess both properties, suggesting that PC1 represents a new paradigm, as a *hybrid* metabotropic-ionotropic receptor-channel protein. As shown in Figure 5, PC1 and PC2 are envisioned to form a heteromeric four-subunit channel complex comprised of three subunits of PC2 and one subunit of PC1 (Yu et al., 2009; Zhu et al., 2011; Wang et al., 2019). In this model, PC1 acts as the fourth subunit of the channel while also functioning as a ligand-activated or mechanosensitive ionotropic receptor that transduces a signal to the channel subunits through PC1-dependent heterotrimeric G protein activation (Parnell et al., 2018). In addition, PC1 may also act as a ligand activated or mechanosensitive metabotropic receptor that can directly activate other downstream signaling events through heterotrimeric G protein activation.

Is there a precedent for metabotropic-ionotropic receptor coupling? One example is glutamatergic signaling in the central nervous system where ionotropic glutamate receptors (iGluRs) and metabotropic glutamate receptors (mGluRs) function in concert (Reiner and Levitz, 2018). There are 18 known iGluRs comprising the AMPAR, KAR, Glud, and NMDAR families and 8 different mGluRs divided into Groups I, II, and III. Both types of receptors multimerize and both bind glutamate in their ligand binding domains. This iGluR and mGluR coupling may result from a direct interaction between their C-terminal tails and/or through scaffolding proteins. It is also likely that there is crosstalk between their respective downstream signaling effectors. For example, iGluRs and mGluRs can cooperate to activate Ca^2+^ signaling through different mechanisms, where iGluR causes Ca^2+^ influx while mGluR causes ER Ca^2+^ release to raise intracellular Ca^2+^. In comparison, PC1 and PC2 may function together more intimately as one multi-subunit complex to regulate Ca^2+^ signaling through distinct but complementary mechanisms. Where the iGluRs and mGluRs segregate these coordinated metabotropic and ionotropic functions in different protein complexes, the PC1 protein may function to carry out both metabotropic and ionotropic functions as a single subunit of the PC1/PC2 receptor-channel complex, where both GPCR and cation channel functions are intrinsic to the PC1 subunit.

Another example of metabotropic-ionotropic receptor coupling involves the Latrophilin/CIRL adhesion GPCR family (Johnson, 2017; Scholz et al., 2017). In this case, two different receptors, the CIRL adhesion GPCR and the NOMPC ion channel cooperate to sense and respond to the same signal–mechanical stress. In neuronal cells expressing these proteins, the NOMPC membrane channel lies anchored to the cytoskeleton and thus is poised to sense and respond to extracellular mechanical forces transmitted through cytoskeletal mechanisms. The 7-TM CIRL protein senses mechanical forces through its extensive extracellular domain interacting with the ECM, transmitting mechanical signals that activate Gαi, which then inhibit adenylate cyclase and lower cAMP to modify the channel function of NOMPC. Thus, in this case two separate proteins, an adhesion GPCR and an ion channel, coordinate the cellular response to mechanical force. In contrast, PC1 alone, as an adhesion GPCR and ion channel subunit may be able to carry out both metabotropic and ionotropic functions as one subunit of the heterotetrameric PC1/PC2 receptor-channel complex.

## 6 Conclusions and future directions

It is of high priority to determine how PC1 functions, including whether PC1 responds to ligand binding or mechanical forces, or both. PC1 contains multiple potential binding motifs that could engage in ligand binding or that could interact with the ECM. At present, all known adhesion GPCRs have 7-TM domains (typical of all canonical GPCRs) and are thought to undergo intracellular “cis” signaling. Cis receptors signal within the same cell on which the receptor resides. Adhesion GPCRs also have additional GPCR-independent trans-cellular “trans” functions mediated by interactions with adhesion receptors on other cells, such as integrins or teneurins (Dunn et al., 2019; Sando et al., 2019; Li et al., 2020; Sreepada et al., 2022). Trans receptors signal by binding receptors on other cells. Thus, it is possible that PC1, as an adhesion GPCR, also does both, and it will be important to dissect these functions and determine the cell and tissue context for each of these multiple possible signaling modalities.

A related question is whether PC1 always functions as an integral subunit of the heteromeric PC1/PC2 receptor-channel complex, or whether it also functions separately as an isolated adhesion GPCR to carry out a PC1-specific signaling role in all cells or in a more limited tissue-specific or developmental context. While it is likely that PC2 can function as a homomeric channel without PC1, it seems less likely that PC1 can function alone, given that the C-terminal 6-TM channel-forming and TOP domains might need to interact with PC2 subunits. However, arguing against this are the many studies where over-expressed PC1, C-terminal domain, or C-tail fragments of PC1 have been shown to activate G protein signaling in a constitutive manner (Delmas et al., 2002; Parnell et al., 2002; Puri et al., 2004) and additional work supporting PC2-independent functions of PC1 (Viau et al., 2020).

As a final thought, it will be informative to examine PC1 in the broader context of the PC1 (and PC2) orthologs. There are four known *PKD1* family paralogs in addition to *PKD1* (PC1). These are human *PKDREJ*, *PKD1L1*, *PKD1L2*, and *PKD1L3*, which unlike *PKD1* have restricted tissue expression (Gunaratne et al., 2007; Kashyap et al., 2019). The protein products of all four are predicted to have 11 TM domains with the last 6 TM domains having ion channel homology. Two undergo GPS cleavage, the exceptions being the products of *PKD1L1* and *PKDREJ*, which do not have the conserved GPS HL^T^/_S_ tripeptide sequence (Butscheid et al., 2006; Field et al., 2011). Both products of *PKD1L1* and *PKD1L2* appear to bind G proteins and thus may have GPCR function (Yuasa et al., 2004), and the *PKDREJ* protein is reported to modulate G protein signaling (Sutton et al., 2006). In addition, there are ten sea urchin (*S. purpuratus*) REJ domain-containing proteins (Gunaratne et al., 2007), some with 11 TM domains that include 6-TM ion channel homology and a GPS cleavage site. Interactions are known to occur between the PC1-like and PC2-like proteins. For example, PC2L1 forms ion channels with both PC1 and PC1L2 (Murakami et al., 2005; Bui-Xuan et al., 2006; Petracca et al., 2016). Important functions for these complexes include left-right asymmetry development in the early embryo (Field et al., 2011; Kamura et al., 2011); formation of a calcium permeable channel on primary cilia (DeCaen et al., 2013); and an unidentified but important role in sour taste perception (Huang et al., 2006; Ishimaru et al., 2006; LopezJimenez et al., 2006). In addition, the complex formed by PC1L3 and PC2L1 shares similar assembly mechanisms and ion channel function as the PC1/PC2 complex (Yu et al., 2012), suggesting that the other PC1-like proteins may also have intrinsic channel function. Taken together, these observations suggest that the PC1 family of bi-functional receptor ion-channel proteins will undoubtedly be found to have many unique biological roles during development, and in tissue and organ physiology, and in human disease.
